# Childhood maltreatment and disrupted parenting in mothers exposed to intimate partner violence: Mediation via mothers' posttraumatic stress symptoms?

**DOI:** 10.1002/imhj.70114

**Published:** 2026-08-13

**Authors:** Willemien M. van den Dorpel, Lenneke R. A. Alink, Carlo Schuengel, Lude J. Rozema, Ralph C. A. Rippe, Anja van der Voort, Sabine van der Asdonk

**Affiliations:** ^1^ Institute of Education and Child Studies Faculty of Social and Behavioral Sciences Leiden University Leiden the Netherlands; ^2^ Clinical Child & Family Studies Faculty of Behavioural and Movement Sciences Vrije Universiteit Amsterdam the Netherlands

**Keywords:** Bayesian analysis, childhood maltreatment, parenting, PTSD, trauma

## Abstract

Parents who have experienced childhood maltreatment may develop posttraumatic stress (PTS) symptoms and display disrupted parenting behavior, particularly in situations of revictimization through intimate partner violence (IPV). This study investigated (1) the relation between parental childhood maltreatment, PTS symptoms, and disrupted parenting, and (2) whether PTS symptoms mediate the relation between childhood maltreatment and disrupted parenting. A culturally diverse sample of IPV‐exposed mothers participated (*N* = 52; *M*_age_ = 30.6 years) and their children (*M*_age_ = 2.8 years; range = 7.5 months–7.0 years), residing in Dutch women's shelters. Multiple Bayesian regression analyses were carried out, and prior probability distributions (priors) were used, informed by a systematic literature search. Results showed a positive association between childhood maltreatment and PTS symptoms but not between these variables and the overall construct of disrupted parenting. No meaningful mediation was found. Exploratory analyses showed some inconclusive evidence for mediation of PTS symptoms in the associations between'childhood maltreatment on the one hand and *role/boundary confusion* and *fearful/disoriented* parenting behavior on the other hand. The findings highlight the relevance of childhood maltreatment in the experience of PTS symptoms. Furthermore, they underscore the need for treatment focused on trauma and parenting in families exposed to IPV.

**Clinical trial registration**: Ethical approval was given by the Medical Research Ethics Committee Leiden The Hague Delft (MREC LDD, protocol number NL74114.058.20) and the study was pre‐registered in the Open Science Framework (DOI: 10.17605/OSF.IO/9EW7P).

## 1 INTRODUCTION

Parents who experience intimate partner violence (IPV) are at risk for parenting problems (Sousa et al., 2022) and posttraumatic stress (PTS) symptoms (Lagdon et al., 2014). Prior to the experience of IPV, many of these parents experienced childhood maltreatment (Herrenkohl et al., 2022; Li et al., 2019; Madigan et al., 2019), which has also been repeatedly found to be associated with PTS symptoms (Gallagher et al., 2022; Lünnemann et al., 2019). Furthermore, childhood maltreatment experiences might affect the *likelihood* of developing PTS symptoms after a later traumatic experience. In other words, they may increase the vulnerability of people to later adverse consequences (McLaughlin et al., 2010). These PTS symptoms in turn could be the cause of suboptimal parenting, including disrupted parenting behavior (Burtchen et al., 2022), which poses a risk for negative child outcomes, such as a disorganized attachment relationship and socioemotional problems (Madigan et al., 2024). To shed more light on the mechanisms underlying disrupted parenting, the current study investigated how childhood maltreatment experiences, PTS symptoms, and disrupted parenting behavior were interrelated. In addition, we examined whether parental PTS symptoms mediated the relation between childhood maltreatment experiences and disrupted parenting.

### 1.1 Parental PTS symptoms and disrupted parenting

PTS symptoms may develop after a traumatic experience such as IPV or child maltreatment. The symptoms include re‐experiencing the traumatic experience, avoiding memories related to the experience, and having negative cognitions (American Psychiatric Association, 2022). The relation between PTS symptoms and various forms of caregiving, as well as the relation between childhood maltreatment and different forms of caregiving have been evaluated in previous studies (Christie et al., 2019; Savage et al., 2019). Direct evidence regarding the relation with disrupted parenting behavior specifically is, however, less available. Moreover, research on this relation in a sample of parents who had all been exposed to (severe) IPV has, to our knowledge, not been conducted at all. Disrupted caregiving behavior is defined as behavior that is atypical and inexplicable or even alarming for children, such as frightened, frightening, or intrusive behavior (Bronfman et al., 2009–2014). Such parenting behavior is seen more often in traumatized parents and is thought to be common in the type of parent‐child interactions that underly disorganized attachment strategies in children (Haltigan et al., 2019; Madigan et al., 2024). The current evidence on the association between parental PTS symptoms and disrupted parenting behavior shows mixed results. Whereas Burtchen et al. (2022) found a positive association between parental PTS symptoms and disrupted parenting, others did not (see e.g., Guyon‐Harris et al., 2021; Schechter et al., 2008), even though they all examined samples of mothers who were severely traumatized, for example by childhood maltreatment and other forms of violence, among which IPV‐exposure. In addition, disrupted parenting is a relatively new construct, and the Atypical Maternal Behavior Instrument for Assessment and Classification (AMBIANCE), a measure designed to measure disrupted parenting, has not been widely used in this research area. The current study therefore further investigated the relation between parents’ PTS symptoms and the level of disrupted caregiving towards their children, as measured with the AMBIANCE, in a sample of IPV‐exposed families.

**Key findings**

1. Childhood maltreatment experiences were related to current PTS symptoms in IPV‐exposed mothers.
2. Childhood maltreatment experiences and PTS symptoms were not related to disrupted parenting as an overall construct, and PTS symptoms did not mediate the relation between childhood maltreatment and disrupted parenting.
3. PTS symptoms were weakly associated with *role/boundary confusion* and *fearful/disoriented* parenting behavior.

**Statement of relevance**

This study examined associations between childhood maltreatment, PTS symptoms, and disrupted parenting, and tested whether PTS symptoms mediated the relation between childhood maltreatment and disrupted parenting. Childhood maltreatment experiences were uniquely associated with adult PTS symptoms among mothers recently exposed to severe intimate partner violence. Mothers residing in women's shelters experienced high levels of PTS symptoms and disrupted parenting, which underscores their urgent treatment needs. The Bayesian framework offers a valuable approach for addressing statistical power issues in small‐sample research.

### 1.2 Parental childhood maltreatment experiences and PTS symptoms

Experiencing child maltreatment as well as victimization of IPV are considered forms of complex trauma, based on their interpersonal and often chronic nature. Compared to other types of traumata, experiences of complex trauma are associated with an even higher risk of different forms of psychopathology, including PTS symptoms (Norman et al., 2012; Pill et al., 2017). Childhood maltreatment experiences have been associated with the experience of PTS symptoms years later, even in adulthood (Messman‐Moore & Bhuptani, 2017). Specifically, individuals who experienced IPV in addition to earlier experiences of childhood maltreatment showed more mental health problems such as PTS symptoms compared to people who only experienced IPV (Lünnemann et al., 2019).

### 1.3 The current study

**FIGURE 1:**
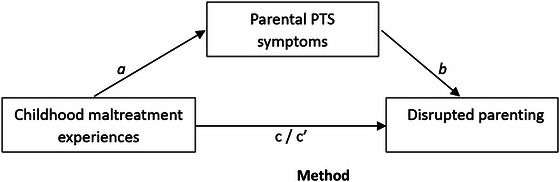
Illustration of the mediation model under study.

What has yet to be investigated, however, is how PTS symptoms play a role in the relation between childhood maltreatment experiences and disrupted parenting. If parents who have experienced childhood maltreatment have an increased risk of developing PTS symptoms and the experience of more PTS symptoms is related to more negative and disruptive parenting behavior, PTS symptoms might mediate the association between childhood maltreatment and subsequent disrupted parenting. The goal of this study was therefore to examine (1) whether mothers with more severe childhood maltreatment experiences reported more severe PTS symptoms and (2) showed more disrupted parenting, and (3) whether mothers who reported more PTS symptoms showed more disrupted parenting behavior towards their children. Subsequently, we investigated (4) whether the relation between childhood maltreatment experiences and disrupted parenting behavior was mediated by the level of PTS symptoms (see Figure 1). Testing these associations contributes to the small evidence base on factors that are possibly linked to disrupted parenting and helps clarify the direction of these associations. Testing the mediating pathway may suggest avenues to breaking the cycle of adverse parenting (Madigan et al., 2019). This is particularly important in IPV exposed parents, because of their increased vulnerability to develop PTS symptoms and experience parenting problems.

To build expectations based on prior research, the current study used a Bayesian modeling approach in the analyses, capitalizing on existing prior knowledge that was obtained through a systematic literature search.

## 2 METHODS

### 2.1 Sample

Participants in this study included 52 mothers (*M*_age_ = 30.6 years, *SD*_age _= 5.8 years, *range* = 18.1 – 48.4 years) and their children (*M*_age_ = 2.8 years, *SD*_age _= 1.8 years, *range* = 7.5 months – 7.0 years; 59.6% boys) who had experienced IPV and received shelter care. Of the mothers, 71.2% (*n = *37) had an educational level lower than ISCED 4 and 90.4% were unemployed at the time of inclusion (*n *= 47). None of the mothers had a cohabitating partner, and they were all the sole caregiver for the child. About one third of the mothers were born in the Netherlands (*n *= 19; 36.5%), almost half were born in Africa, Latin America, or Turkey (*n *= 24; 46.2%), and the remainder were born in Europe (other than the Netherlands) or Indonesia (*n *= 9; 17.3%). The small sample formed an additional reason to pursue Bayesian analyses to address power issues.

The dyads were recruited from two Dutch organizations that offer shelter for families after domestic violence. They could be included if (1) the dyad resided in the domestic violence shelter due to IPV or the dyad received ambulatory care from the shelter after the safety in the family situation had been classified as highly unsafe (‘code red’) and specific safety measures were taken (a restraining order for the (ex‐)partner or an alarm system), (2) the child was between 6 months and 6 years old at the moment of inclusion (if there was more than one child in this age range in the family, the youngest child between 1.5 and 6 years old was selected for participation, except in cases where the non‐residing parent did not give permission for this child and did not have custody over another child in the family who fit the criteria), and (3) the parent spoke Dutch or English sufficiently to fill out the questionnaires or a professional interpreter was available.

Residing parents with a serious mental illness (other than PTS) that required immediate intervention were excluded from participation. In addition, if the father who had custody of the child was not allowed to know the child's place of residence due to safety reasons, the dyad was also excluded because in this case, the required permission of the father could not be obtained. For one dyad, the children in the age category were twins, and so both of them participated. To avoid dependency in the data, only the data of one of them were analyzed.

### 2.2 Procedure

Data for this study were collected as part of a pre‐test measure for a randomized controlled trial (see Van der Asdonk et al., 2022 for the study protocol). A total of 105 parents were approached by their social worker for participation, who gave them a flyer or told them about the study. Of these, 24% (*n *= 25) did not want to receive more information about the study and were excluded. For the remaining parents, all information about the study was provided during a conversation with one of the researchers, after which 12% (*n *= 13) declined to participate. For 14% (*n *= 15) of the families, the mother consented but the other custodial parent (i.e., the father) did not. The remaining 50% (*N *= 52) of families gave consent and were included and participated in the pre‐test.

The pre‐test consisted of a 1‐hour appointment at the shelter location or the parent's home in case they received ambulatory care. During the appointment, parents filled out a questionnaire to report on their own mental health and the mental health of their child. The questionnaires were filled out in Dutch for the Dutch‐speaking participants. Non‐Dutch‐speaking participants filled out the questionnaires in their native language if a validated version was available. Otherwise, they completed the questionnaires in English, provided they had sufficient proficiency. For the participants who preferred so, a translator assisted via phone during the research appointments. A considerable number of participants spoke Arabic as their first language and thus all questionnaires were made available in Modern Standard Arabic. After completing the questionnaires, parents participated together with their child in a task that was videotaped. First, the dyad played for 5 min without any toys, and then they received a box full of toys that they could play with for 4 min. Lastly, they had 1 minute to clean up all the toys. Participants received compensation of 20 euros for the 1‐hour appointment. Ethical approval was given by the Medical Research Ethics Committee Leiden The Hague Delft (MREC LDD, protocol number NL74114.058.20) and the study was pre‐registered in the Open Science Framework (DOI: 10.17605/OSF.IO/9EW7P).

### 2.3 Measures

#### 2.3.1 Demographic characteristics

During pre‐test, parents filled out a questionnaire about demographic characteristics, such as age, gender, employment, education level, parenthood status (single parent or not), and country of birth. Single parenthood was coded as such when children lived fulltime with the participating parent and did not reside with the other parent.

#### 2.3.2 Lifelong exposure to traumatic experiences

To be able to give an overview of the number of traumatic experiences of the participants, we used the Life Events Checklist (LEC‐5; Blake et al., 1995), which measures exposure to traumatic experiences that include a so‐called Criterion A for Posttraumatic Stress Disorder (PTSD) (American Psychiatric Association, 2022). Next to the Dutch and English version of the LEC‐5, validated versions in Arabic, Polish, and Turkish were used. The list consists of 17 items each describing a potentially traumatic event, such as natural disasters, war, and severe injuries. For every item, participants indicated if and how they were exposed to the traumatic experiences, namely whether they: (1) experienced it themselves, (2) witnessed it happen, (3) heard about it happening to a close family member or friend, (4) were exposed to it in relation to occupational activities, (5) were not sure if they experienced it, or (6) did not experience it. We used a count score for the number of different types of traumatic experiences a person was exposed to, where options (1) and (2) were coded as a yes, as the other options were more indirect types of experiences. The goal of this was to obtain a conservative estimate of the experience of traumatic events. It was not possible from the results of the LEC‐5 to distinguish between traumatic childhood experiences and other traumatic experiences and therefore controlling for this variable in the main analyses was not deemed informative.

#### 2.3.3 Childhood maltreatment experiences

All parents reported their childhood maltreatment experiences on the short version of the Childhood Trauma Questionnaire (CTQ‐SF; Bernstein et al., 2003). The questionnaire is widely used to measure retrospective childhood maltreatment and has been validated in various samples (Georgieva et al., 2021). The CTQ consists of five subscales (emotional abuse, physical abuse, sexual abuse, emotional neglect, and physical neglect), each consisting of five items, except the sexual abuse scale, which in the Dutch version has four items (the item *‘Someone molested me’* was omitted; Thombs et al., 2009). Each item constitutes a specific statement, for example ‘*I didn't have enough to eat*’ or ‘*I got hit so hard by someone in my family that I had to see a doctor or go to the hospital*’. In addition, there are three minimization/denial items. For each item, participants rated on a scale of 1–5 (*never true—very often true*) how often the statement was true in their experience. The total sum score was used in the analyses (α in the current sample for all items = .93), indicating the severity of childhood maltreatment experiences, where a higher score indicated a higher severity. If single items were missing (14 missing items from six participants), these were imputed with the mean score of the participant on the specific subscale of the questionnaire that the missing value was part of. To estimate the prevalence of the different subtypes of childhood maltreatment within the current sample, the cut‐off scores for the Dutch version were used (Thombs et al., 2009).

Next to the Dutch and English version of the CTQ‐SF, validated versions in Polish and Turkish were used. A native Arabic‐speaker and a professional translator created a translated version of the Childhood Trauma Questionnaire Short Form (by means of back‐and‐forth translation).

#### 2.3.4 Parental PTS symptoms

The PTSD checklist for the DSM‐5 (PCL‐5; Blevins et al., 2015; Dutch version: Hoeboer et al., 2024) was used to measure parental PTS symptoms. In addition to the Dutch and English versions of the PCL‐5, validated versions in Arabic, Polish, and Turkish were used. The questionnaire measures the severity of 20 PTS symptoms that correspond to the criteria for PTSD symptoms in the DSM‐5. Every item was measured on a scale of 0–4 (*not at all—extremely*). The sum of all symptoms was used in the analyses (Cronbach's *α* in the current sample for all items: .94, assuming unidimensionality). If single items were missing (seven items from six participants), these were imputed with the mean score of the parents on this questionnaire. The PCL‐5 has been found reliable with good concurrent validity with other measures for PTS symptoms (Forkus et al., 2022).

#### 2.3.5 Disrupted parenting behavior

Video recordings of the parent‐child interactions were coded using the Atypical Maternal Behavior Instrument for Assessment and Classification (AMBIANCE; (Bronfman et al., 2009–2014). For feasibility, and in consultation with one of the AMBIANCE developers, only the last 2 min of the play session without toys, and the full play session with toys (5 min) were coded, as a single fragment of 7 min. Videos from participants who did not speak English or Dutch were translated by native speakers of the spoken language, after which the videos were coded. With the AMBIANCE, the presence of 150 specific behaviors displayed by the parent to the child are scored. There are five dimensions (i.e., affective communication errors, role/boundary confusion, fearful/disoriented behaviors, intrusiveness/negativity, and withdrawal) that receive a score between 1 and 7, where higher scores indicate higher levels of disruption. Based on these subdimensions, one overall score is assigned to classify parents’ behavior from 1 (*high normal*) to 7 (*highly disrupted*). The parent‐child interaction is considered disrupted if an overall score higher than 4 is assigned (Bronfman et al., 2009–2014). The overall scores were used in the main analyses, and the scores for the subdimensions for explorative analyses. Coding of the videos was performed by two independent coders, one of whom is a developer of the AMBIANCE and the other one a certified coder. Together, the coders achieved a good interrater consistency, as quantified through the intraclass correlation (ICC = .85 for the overall score; absolute agreement, single measures) on a set of 32 randomly selected video recordings from the dataset as collected according to the study protocol for the RCT (see Van der Asdonk et al., 2022). The coders also achieved good reliability on all subscales, except for the withdrawal subscale (ICC = .72 for *affective communication errors*; ICC = .84 for *role/boundary confusion*; ICC = .71 for *fearful/disoriented behaviors*; ICC = .79 for *intrusiveness/negativity*; ICC = .65 for *withdrawal*). To prevent coding drift, the coders regularly met to discuss video recordings for which they were unsure about the scores.

### 2.4 Analyses

To analyze the relation between childhood maltreatment experiences, PTS symptoms, and disrupted parenting behavior and to examine whether PTS symptoms mediated the relation between childhood maltreatment experiences and disrupted parenting behavior, we used the statistical analysis program *R* (Version 4.4.2; R Core Team, 2024). First, all variables were standardized. Then, three separate Bayesian regression analyses were performed to inspect the total relation between childhood maltreatment experiences and disrupted parenting (*c*, see Figure 1) and the simple relations between childhood maltreatment experiences and PTS symptoms (the *a* path in the mediation model, see Figure 1) and between PTS symptoms and disrupted parenting behavior. After that, a separate analysis was carried out to analyze the direct relation between PTS symptoms and disrupted parenting, while accounting for childhood maltreatment (the *b* pathway in the mediation model, see Figure 1). Subsequently, the indirect relation between childhood maltreatment experiences and disrupted parenting via the level of PTS symptoms was estimated, computed as the product of the posterior regression coefficients of path *a* and path *b* (see Figure 1).

To interpret the substantive meaning of the posterior regression coefficient a Region of Practical Equivalence (ROPE) was defined, which ranged from −.10 to .10 (Kruschke, 2018). This is the region that defines the range of non‐meaningful effects. For every specified model, the 95% posterior probability interval (PPI, similar to a confidence interval in frequentist statistics) was interpreted in relation to the ROPE. In addition, the Bayes Factor (BF) was calculated to compare different models to each other, to estimate the probability of a mediation effect. A higher BF indicated more evidence for a certain model.

#### 2.4.1 Prior specification

For each of the paths under study, a prior distribution for the regression slopes was defined, based on prior information about this relation that was reported in the literature. A systematic literature search was performed and a total of 27 papers were found (see Supporting Information: Appendix A for details). To define a prior that is based on samples that are as close as possible to that of the current study, the set of included papers was classified into papers with study samples with a relatively high level of demographic risk and study samples without a high level of demographic risk. The decision to select samples on high demographic risk was made because demographic factors, such as employment, education, relationship status, and income level, can affect the risk of parenting problems and child maltreatment (see e.g., Madigan et al., 2023). Differentiation based on level of demographic risks could thus prevent biased prior specification.

The means and standard deviations of the prior distributions for every pathway were obtained from aggregation of the available literature on that specific pathway. For sensitivity analyses, a total of three priors were specified, such that for every pathway an informative prior (based on the papers on high‐risk samples), a weakly informative prior (based on the findings of all 27 included studies), and a diffuse or uninformative prior (using broadly specified priors with a mean of zero and SD of .5) were specified. To identify which prior distribution resembled the data best, the Bayes Factor was used to compare the model parameters resulting from different prior distributions. A higher Bayes factor was expected for the model results estimated with the informative prior, compared to the model results estimated with the less informative priors. This would indicate that the data fit best under the informative prior distributions.

Table 2 shows the three operationalizations of the priors for each path in the model. The prior distribution was only specified for the regression slopes, for the other parameters the default non informative priors as used in *Brms* (version 2.22; Bürkner, 2017) were used. Based on the systematic literature search, we believe that the posterior regression coefficient was within the prior range, and there was no reason to assume a different probability for values that were (much) higher or lower than the specified prior range (i.e., symmetry was assumed). All prior distributions were thus specified as normal distributions (see Table 1). The results of the prior convergence checks of the regression coefficients, the prior sensitivity analyses, and the outcomes of the regression models are reported according to the When to worry and how to Avoid the Misuse of Bayesian Statistics (WAMBS) checklist (Depaoli & van de Schoot, 2017). A filled‐out version of the checklist can be found in the Supporting Information 1.

**TABLE 1:** Normal prior specification for the Bayesian models under study.

Pathway	Type of prior	*M*	*SD*
Childhood maltreatment → PTS symptoms	Most informative	.31	.09
	Weakly informative	.35	.14
	Diffuse	0	.50
PTS symptoms → disrupted parenting	Most informative	.30	.13
	Weakly informative	.24	.13
	Diffuse	0	.50
Childhood maltreatment → disrupted parenting	Most informative	.16	.10
	Weakly informative	.12	.12
	Diffuse	0	.50

#### 2.4.2 Model fitting and prior convergence checks

For every pathway under study, a regression model was set up with four chains of 20,000 iterations, of which 10,000 were burn‐in iterations, using Markov Chain Monte Carlo sampling. Convergence of the model was checked using the Gelman and Rubin convergence diagnostic (also referred to as Rhat or Potential Scale Reduction Factor). If this value is within ± .05 of 1, convergence is good (Gelman & Rubin, 1992).

To check for the possibility of local convergence, and thus a suboptimal model (i.e., in the model with the 20,000 iterations), models were also estimated with double the number of iterations (40,000 with 20,000 burn‐in iterations). Over this model with double the number of iterations, the Geweke convergence diagnostic was then calculated, to assess the stability of the chains. It compares the first half of the chain with the second half using a *z*‐test. If the *z*‐statistics that are produced are higher than 1.96, the chains do not converge because the first half is significantly different from the second half. Further convergence checking was done by calculating the relative deviation between the regression coefficients of the different models (with and without double the number of iterations), and this was interpreted in light of the substantive meaning of the coefficients. The substantive meaning of the relative deviation between the two models had to be negligible; otherwise, results were not reported.

Before interpreting the posterior model results, different plots were visually inspected to check: (1) whether the histogram of the different parameters within the posterior model was smooth and did not contain abnormalities (such as gaps), indicating that the posterior model contained enough information, (2) whether the autocorrelation plots showed that the different chains contained a comparable amount of autocorrelation and that the degree of autocorrelation was generally low, indicating good convergence among the different chains, (3) whether the posterior Kernel density plot was single peaked and, (4) whether the posterior distribution made substantive sense, as indicated with the posterior SD and the 95% PPI both being within the range of the original regression coefficient (β; −1 to 1). The different plots for the main analyses can be found in the Supporting Information (Tables 1, 2, 3), as part of the WAMBS‐checklist.

**TABLE 2:** Baseline characteristics of participants (*N* = 52).

Baseline measures	*M*	*SD*	range
PTS symptoms parent (PCL‐5)	36.7	18.5	0–72.6
Childhood maltreatment (CTQ‐SF)	50.1	20.6	25–102.8
Number of traumatic experiences (LEC‐5)	5.4	3.0	0–14
Disrupted caregiving (AMBIANCE)	4.0	1.8	1–7
D1: Affective communication errors	3.8	1.8	1–7
D2: Role/boundary confusion	2.9	1.4	1–7
D3: Fearful/disoriented behaviors	2.8	1.7	1–6
D4: Intrusiveness/negativity	4.2	1.7	1–7
D5: Withdrawal^a^	2.0	1.4	1–6

^a^ Could not be coded reliably due to low prevalence in this sample, so should be interpreted with caution.

**TABLE 3:** Correlations for background variables and study variables (*N* = 52).

Variable	1	2	3	4	5	6	7	8	9	10	11
1. Age mother	—										
2. Age child	.46^**^	—									
3. Gender child^a^	‐.19	‐.10	—								
4. Number of traumatic experiences	.00	.08	.10	—							
5. Childhood maltreatment experiences	.07	‐.09	‐.13	.33^*^	—						
6. PTS symptoms	.04	.13	.21	.31^*^	.26	—					
7. Total disrupted parenting	‐.24	‐.42^**^	‐.16	‐.16	‐.18	‐.05	—				
8. Affective communication errors	‐.14	‐.35^**^	‐.12	‐.14	‐.11	.00	.90^**^	‐			
9. Role/boundary confusion	‐.21	‐.28^*^	‐.08	‐.12	‐.11	.11	.69^**^	.72^**^	–		
10. Fearful/disoriented behaviors	‐.21	‐.34^**^	.14	‐.13	‐.18	.09	.70^**^	.61^**^	.52^**^	–	
11. Intrusiveness/negativity	‐.23	‐.38^**^	‐.10	‐.12	‐.29^*^	‐.09	.89^**^	.86^**^	.65^**^	.58^**^	–
12. Withdrawal	‐.14	‐.32^*^	‐.02	‐.10	‐.07	‐.01	.47^**^	.27^*^	.07	.57^**^	.23

^a^ 0 = boy 1 = girl.

* *p* ≤ .05.

** *p* ≤.01.

Results are reported for the models with 20,000 iterations with 10,000 burn‐in iterations, provided that: (1) they had reached convergence, as indicated by the Gelman and Rubin convergence diagnostic, (2) they did not indicate signs of local convergence (but rather overall convergence), (3) the histogram, autocorrelation plot and Kernel density plot did not show any abnormalities, and (4) the posterior distribution made substantive sense. If results are not reported for the model with 20,000 iterations but for a model with more iterations (e.g., if there were indications for local convergence after doubling the number of iterations), this is indicated in the results.

## 3 RESULTS

### 3.1 Baseline characteristics

Baseline measures showed that 61.5% of mothers (*n *= 32) had experienced at least one form of childhood maltreatment according to the moderate‐to‐severe cut‐off of the CTQ‐SF (Bernstein et al., 2003). This was 82.7% (*n = *43) when using the low‐to‐moderate cut‐off. That means that only 9 participants (17.3%) had experienced none‐to‐minimal childhood maltreatment. Mothers had experienced, on average, five traumatic experiences throughout their life (*SD *= 3). In terms of PTS symptoms, 67.3% (*n *= 35) of mothers scored higher than the internationally recommended cut‐off of 31 (Forkus et al., 2022), which increased to 78.8% when using the cut‐off of 22, for the Dutch population (Hoeboer et al., 2024). Table 1 shows all baseline measures of the participants.

Table 3 shows correlations between the background and study variables. Of note, age of the child correlated negatively with disrupted parenting and was therefore included as covariate in the analyses. In addition, there was a negative correlation between childhood maltreatment and intrusiveness. No other control variables than “age of the child” were included in the analyses, as none of the other variables correlated strongly with the outcome measure (see Table 3).

### 3.2 Childhood maltreatment as a predictor of disrupted parenting

Results of the analyses using the informative prior (∼*N*(.16, .10)) showed that the posterior regression coefficient (β) was estimated at .02 (*posterior SD *= .08). The 95% PPI was between −.14 and .18. The PPI was for 80.7% within the ROPE. This indicates that, for the model incorporating the informed prior, there was a posterior probability of 19.3% that the relation between childhood maltreatment and disrupted parenting was meaningfully different from zero.

To evaluate the influence of the prior that was chosen on the results that were obtained, the analyses were repeated with a weakly informative prior (∼*N*(.12, .12)) and a diffuse prior (∼*N*(0,.5)) for the regression coefficient, see Table 4. Comparison with the weakly informative prior showed that the relative deviation between the estimated regression coefficients in the two models was 281.4%. The results were not substantively different (*β *= −.04, *posterior SD *= .09, PPI: −.21–.14), as the regression coefficient was still close to zero, but it became negative. The overlap between the PPI and the ROPE was 72.9%, indicating a probability of 27.1% that the relation is meaningfully different from zero according to the model with the weakly informed prior. The Bayes Factor in relation to the model incorporating the informative prior was .46, which meant that the data fit better under the model with the weakly informative prior than under the model with the informative prior.

**TABLE 4:** Results of the Bayesian regression analyses for each of the specified priors.

Type of prior	Predictor	*β*	*SD*	95% PPI		Percentage in ROPE
				LL	UL	
Childhood maltreatment → disrupted parenting						
Informative	Childhood maltreatment Age child	.02 ‐.23	.08 .07	‐.14 ‐.38	.18 ‐.08	80.65 1.45
Weakly informative^a^	Childhood maltreatment Age child	‐.04 ‐.23	.09 .07	‐.21 ‐.38	.14 ‐.09	72.93 .63
Diffuse^a^	Childhood maltreatment Age child	‐.21 ‐.24	.13 .07	‐.45 ‐.38	.04 ‐.10	17.26 0
Childhood maltreatment → PTS symptoms						
Informative	Childhood maltreatment	.29	.08	.14	.44	0
Weakly informative^a^	Childhood maltreatment	.30	.10	.11	.50	0
Diffuse	Childhood maltreatment	.24	.13	‐.02	.50	12.52
PTS symptoms and disrupted parenting						
Informative	PTS symptoms Age child	.16 ‐.24	.09 .07	‐.03 ‐.39	.34 ‐.10	26.19 .27
Weakly informative	PTS symptoms Age child	.13 ‐.24	.09 .07	‐.06 ‐.39	.31 ‐.09	38.39 .41
Diffuse	PTS symptoms	.01	.13	‐.25	.26	59.08
	Age child	‐.23	.07	‐.38	‐.09	1.26
Childhood maltreatment → Disrupted parenting, via PTS symptoms						
Informative^a^	Indirect effect	.05	.03	‐.01	.11	97.26
Weakly informative	Indirect effect	.04	.03	‐.01	.11	97.46
Diffuse	Indirect effect	<.01	.04	‐.09	.08	100

*Note*: *SD* refers to the posterior *SD*, *PPI* refers to the 95% PPI.

^a^ Reported for the model with 40,000 iterations.

Comparison of the model with the informative prior with the model with the diffuse prior showed that the relative deviation between the estimated regression coefficients in the models with the informative and the diffuse prior was 1124.8%. The results of the model with the diffuse prior showed a negative regression coefficient (*β *= −.21, *posterior SD *= .13, PPI: −.45–.04). Overall, 17.3% of the PPI was in the ROPE, meaning that there was a probability of 82.7% that this relation is meaningfully different from zero, in a negative direction. The diffuse prior fit the data best, as shown by the Bayes factor that was .22, in favor of the diffuse prior (compared to the model with the informative prior). Taken together, the results suggest that there is inconclusive evidence that the association between childhood maltreatment and disrupted parenting is meaningfully different from zero, that is, outside the ROPE. According to the posterior regression coefficients, the true posterior coefficient likely ranges between −.21 and .02, reflecting the observed range of regression coefficients. This means that there is inconclusive evidence for the hypothesized positive relation between childhood maltreatment and disrupted parenting.

Exploratory analyses showed that the associations between childhood maltreatment experiences and different subdimensions of disrupted parenting were not different from the association with disrupted parenting in general (see Supporting Information: Appendix B for exploratory results). Overall, there was inconclusive evidence for a relation that was meaningfully different from zero (see Table B1 for detailed results). For *intrusiveness/ negativity*, results with the diffuse prior showed an indication for a total negative association. This was only the case for the diffuse prior, however, so results overall remained inconclusive.

### 3.3 Childhood maltreatment as a predictor of PTS symptoms

Results of the analyses using the informative prior (∼*N*(.31, .09)) showed that the posterior regression coefficient (*β*) was estimated at .29 (*posterior SD *= .08). The 95% PPI was between .14 and .44, indicating that there was a 95% probability that the β was within that range. As the PPI was completely outside the ROPE, the *β* likely was meaningfully different from zero.

To evaluate the sensitivity of the prior that was chosen on the results that were obtained, the analyses were repeated with a weakly informative prior (∼*N*(.35, .14)) and a diffuse prior (∼*N*(0,.5)) for the regression coefficient; see Table 4. Comparison with the model resulting from the weakly informative prior showed that the relative deviation of the posterior regression coefficient as estimated in the two models was 4.6%. The results were not substantively different (*β *= .30, *posterior SD *= .10, PPI: .11 ‐ .50). The PPI was thus still outside the ROPE for 100%, which means that the substantive meaning of the results did not change and the β still had a size that was likely meaningfully different from zero. The Bayes factor that was calculated over the two models had a value of 1.3, meaning that the informative prior fit the data somewhat better than the weakly informative prior.

Comparison of the informative prior with the diffuse prior showed that the relative deviation of the posterior coefficient as estimated in the model with the informative and the diffuse prior was 17.2%. The results of the model with the diffuse prior showed a regression coefficient that was a bit smaller (*β *= .24, *posterior SD *= .13, PPI: −.02–.50). Overall, 12.5% of the PPI was within the ROPE, meaning that there was an 87.5% probability that the regression coefficient was meaningfully different from zero. Further model comparison to the informative prior showed that the Bayes factor was 3.2 in favor of the model with the informative prior, meaning that the model under the informative prior fit the data better than the model under the diffuse prior. Taken together, there was an 87.5%–100.0% probability that the relation between childhood maltreatment experiences and PTS symptoms was positive and between .24 and .30, which is meaningfully different from zero. Overall, this means that parents who reported more childhood maltreatment experiences displayed more PTS symptoms.

### 3.4 PTS symptoms as a predictor of disrupted parenting

Results of the analyses with the informative prior (∼*N*(.30, .13)) showed that the regression coefficient (*β*) was estimated at .16 (*posterior SD *= .09). The 95% PPI was between −.03 and .34, indicating a 95% probability that the *β* was within that range. 26.2% of the PPI was within the ROPE. This indicates that there was a probability of 73.8% that this relation was meaningfully different from zero.

To evaluate the sensitivity of the prior that was chosen on the results that were obtained, the analyses were repeated with a less informative prior (∼*N*(.24, .13)) and a diffuse prior (∼*N*(0,.5)) for the regression coefficient. Table 4 shows an overview of all results. Comparison of the model based on the informative prior to the model based on the weakly informative prior showed that the relative deviation between the estimated regression coefficients was 19.3%. The results were not substantively different (*β *= .13, *posterior SD *= .09, PPI: −.06–.31). Overall, 38.4% of the PPI overlapped with the ROPE. This indicates that the probability that the relation was meaningfully different from zero was 61.6% according to the model with a weakly informed prior.

Comparison of the model with the informative prior with the model with the diffuse prior showed that the relative deviation between the estimated regression coefficients was 95.5%. The results of the model with the diffuse prior showed a regression coefficient close to zero (*β *= .01, *posterior SD *= .13, PPI: −.25–.26). Overall, 59.1% of the PPI was within the ROPE, meaning that there was a probability of 40.9% that this relation was meaningfully different from zero. Comparison of the models with the different priors showed that the data fit the distribution under the weakly informed prior the best (*BF *= 1.3, compared to the diffuse prior). The informed prior did thus not lead to the best fitting model; the data even fit the model with the diffuse prior better (*BF *= .83, in favor of the diffuse prior). Taken together, there was a 40.9%–73.8% probability that the relation between PTS symptoms and disrupted parenting was between .01 and .16, which is likely not meaningfully different from zero. This means that there is inconclusive evidence for the hypothesized positive relation between PTS symptoms and disrupted parenting.

Exploratory analysis of the relation between PTS symptoms and the different subdimensions of disrupted parenting showed that there was likely no meaningful relation between PTS symptoms and the subdimensions *affective communication errors* and *intrusiveness/negativity*, as the PPIs for all three different prior specifications consistently included zero (see Supporting Information: Appendix B for exploratory results). The relations between PTS symptoms and *role/boundary confusion* and *fearful/disoriented behaviors* might be more pronounced. PPIs of these separate relations, while controlling for child age, showed that zero was not included in the PPIs of the models with the informed prior and the weakly informed prior. Posterior regression coefficients for the relation between PTS symptoms and *role/boundary confusion* under the models with the diffuse, weakly informative and informative prior ranged from .14 to .23, with the percentages outside the ROPE varying between 63.3% and 93.6%. The data fit the distribution best under the weakly informed prior (*BF *= 2.5, compared to the diffuse prior). The informed prior did thus not lead to the best fitting model, but it still fit the data better than the model using the diffuse prior (*BF *= 2.1). Overall, this suggests that parents who experienced more PTS symptoms showed more *role/boundary confusion* behaviors in communication with their children.

Posterior regression coefficients for the relation between PTS symptoms and *fearful/disoriented behaviors*, under the models with the diffuse, the weakly informative and the informative prior ranged from .13 to .22, with the percentages outside the ROPE varying between 60.6% and 92.0%. Comparison of the models with the different informed priors showed that the model under the weakly informed prior fit the data best (*BF *= 2.4, compared to the diffuse prior), right after the informed prior (*BF* = 1.95). This altogether suggests that parents who experienced more PTS symptoms showed more *fearful/disoriented behaviors* to their children. See Table B2 for detailed results per model.

### 3.5 Mediating role of PTS symptoms in the relation between childhood maltreatment experiences and disrupted parenting behavior

For the mediation model *(ab*), the regression coefficient (*β*) with the informative priors was close to zero (*β *= .05; *posterior SD *= .03). The 95% PPI was between −.01 and .11, and had a 97.2% overlap with the ROPE. This indicated that, according to the model with the informative prior, there was a probability of 97.2% that PTS symptoms did not meaningfully mediate the relation between childhood maltreatment and disrupted parenting.

Checking the sensitivity of the priors by comparing the results with those from the models with the weakly informed priors and the non‐informative priors showed comparable results (Table 4). Comparison with the weakly informative prior showed that the relative deviation of the regression coefficients as estimated in the two models was 13.2%. The results were not substantively different from the model with the informative prior (*β *= .04, *posterior SD *= .03, PPI: −.01–.11). 97.5% of the PPI overlapped with the ROPE, indicating that mediation by PTS symptoms is not likely.

Lastly, results of the informative prior were compared with those of the diffuse prior. The relative deviation between the models was 101.9%, and the regression coefficient in the model with the diffuse prior was negative (*β *< .01, *posterior SD *= .04, PPI: −.09–.08). The PPI had 100.0% overlap with the ROPE, so the substantive meaning did not change: the relation between childhood maltreatment and disrupted parenting is not likely to be mediated by PTS symptoms. This finding was confirmed by the Bayes Factor, which was estimated over the model including the mediator with the model without the mediation. The Bayes Factor for the models with the different priors ranged from .01 to .35, all in favor of the model were disrupted parenting was only predicted by childhood maltreatment experiences, and PTS symptoms were not included in the model. There was thus no meaningful mediation of PTS symptoms in the relation between childhood maltreatment and disrupted parenting as an overall construct.

Exploratory analysis of the mediation model applied to the subdimensions *affective communication errors* and *intrusiveness/negativity* showed similar results, reflecting that there was probably no mediation through PTS symptoms (see Supporting Information: Appendix B for exploratory results). For the subdimensions *role/boundary confusion* and *fearful/disoriented behaviors*, there was minimal evidence for mediation of PTS symptoms, as the PPI of the informative prior and the weakly informative prior did not include zero (see Table B3 for the results). In addition, the Bayes Factor when comparing the full model with the reduced model, under the weakly informed prior, had a value over 1 (*BF_r/b confusion_* = 1.2; *BF_f/d behavior_ *= 1.2), meaning that the data fit the full model including the mediator better compared to the model without the mediator. The size of the mediation effect was probably not meaningfully different from zero, however, as most of the posterior distribution was within the ROPE (> 80% of PPI's were within the ROPE; see Table B3 for detailed results). In addition, the Bayes Factors when comparing the models (i.e., the model with and without the mediator) under the informed priors (*range BF *= .78–.84) and the diffuse priors (*range BF* = .68–.76) were all in favor of the reduced model, where disrupted parenting was only predicted by childhood maltreatment experiences. Altogether, this means that there was some inconclusive evidence that there was an indirect effect of PTS symptoms mediating the relation between childhood maltreatment experiences and *role/boundary confusion* and *fearful/disoriented behaviors*, but the size of the effect was probably negligible.

## 4 DISCUSSION

The goal of the current study was to investigate the relation between childhood maltreatment experiences, PTS symptoms, and disrupted parenting behavior in mothers who have been exposed to IPV. As the current sample was small, the analyses were informed with prior findings about the magnitude of these different relations, using a Bayesian approach. Results suggest that the relation between maternal childhood maltreatment and PTS symptoms was positive and moderate in size. Furthermore, there was no evidence for a meaningful relation between childhood maltreatment experiences and the overall construct of disrupted parenting, nor between PTS symptoms and the overall construct of disrupted parenting, all controlling for children's age. In addition, there was some evidence for a positive relation between PTS symptoms and *role/boundary confusion* and *fearful/disoriented* parenting behavior specifically. There was no evidence that PTS symptoms mediated the association between childhood maltreatment experiences and the overall construct of disrupted parenting.

The expected relation between childhood maltreatment and PTS symptoms was confirmed in the current study. This aligns well with existing literature on the influence of childhood maltreatment on psychological conditions (Baldwin et al., 2023). The current study shows that, even though all participants experienced recent and severe IPV, childhood maltreatment experiences still seemed to contribute uniquely to PTS symptoms. One reason for this could be the vulnerability to adverse consequences that is associated with prior experiences of childhood maltreatment, as was described in the Introduction. At the same time, this could mean that those PTS symptoms already existed for some time and have never been treated. Previous studies show that PTS symptoms might often be overlooked and unrecognized (Da Silva et al., 2019) and that people can face numerous barriers and challenges in the process of seeking help for PTS symptoms (Smith et al., 2020). The current study therefore adds to the current literature by underscoring the necessity of early detection and improving accessibility of treatment for PTS symptoms in IPV exposed parents.

The findings that neither childhood maltreatment experiences nor PTS symptoms were associated with disrupted parenting behavior, and that PTS symptoms did not mediate the relation between childhood maltreatment experiences and disrupted parenting, were contrary to the specified hypotheses. Meta‐analytic evidence shows a positive relation between childhood maltreatment and negative forms of parenting, such as harsh parenting, intrusive parenting, and withdrawing behavior of parents (Savage et al., 2019). One of the reasons for not finding support for this relation might be the relative homogeneity of the current sample regarding their adverse experiences. Except for nine participants, all reported to have experienced at least minimal forms of childhood maltreatment, and most participants experienced at least moderate to severe childhood maltreatment. In addition, mothers had all experienced severe IPV, most of them had experienced various other traumatic experiences, and most faced demographic risk factors. Altogether, this may have confounded the relations under study, for example in the association between childhood maltreatment and disrupted parenting. Demographic risk factors could possibly affect parenting behavior and, with that, child outcomes (Madigan et al., 2023), and it could be that those factors explained more variance in the relation between childhood maltreatment experiences and disrupted parenting than childhood maltreatment alone. This might be one of the reasons why no direct relation between childhood maltreatment and disrupted parenting was detected in another high‐risk sample as well (see e.g., Guyon‐Harris et al., 2021). Controlling for demographic risks, such as unemployment and single parenthood, was not informative in the current sample, however, as almost all participants faced those.

There is an extensive body of literature that shows evidence for a relation between childhood maltreatment experiences and PTS symptoms and indices of negative parenting, but literature that uses the AMBIANCE coding system specifically and the overall construct of disrupted parenting is scarcer. Some studies that used the AMBIANCE did not find a positive association between childhood maltreatment and disrupted parenting behavior, and PTS symptoms and disrupted parenting (see e.g., Guyon‐Harris et al., 2022; Schechter et al., 2008) even though other studies did find a relation in the expected direction (see e.g., Burtchen et al., 2022). Altogether, studies using the AMBIANCE are limited and findings have been mixed. Evidence regarding the sensitivity and specificity of the AMBIANCE in detecting change in disrupted parenting behavior is currently lacking. It could be that the overall 7‐point scale is not nuanced enough to capture subtle differences. Although all seven scale points were assigned, most scores (60%) were between 4 and 6, suggesting limited differentiation. The addition of a more micro‐analytic approach, where specific parenting behaviors are coded in addition to the overall scale and subscales, might provide more nuance in how specific disrupted parenting behaviors are expressed and are related to childhood maltreatment and PTS symptoms. However, such a micro‐analytic addition could introduce other interpretational challenges, as it would move away from the original instrument and other studies.

Alternatively, it is also possible that no relation between childhood maltreatment experiences and parenting and PTS symptoms and parenting existed in the current sample. For example, once childhood maltreatment and PTS symptoms exceed a certain level of severity, their association with parenting may no longer be meaningful. This would indicate a possible ceiling effect, where above very high levels of traumatic experiences, their association with parenting may no longer be present, or detectable, at least not with the measures as used in the current study. The current sample experienced relatively high levels of childhood maltreatment and PTS symptoms, and therefore it was not possible to examine such a threshold. Alternatively, there may be other factors that could indirectly relate PTS symptoms and childhood maltreatment to disrupted parenting, such as other mental health symptoms (Burtchen et al., 2022), or parents’ own attachment representation (Guyon‐Harris et al., 2021; Schechter et al., 2008). Within the scope of the current study, it was not possible to control for all these factors, which can be seen as a limitation.

Exploratory analyses including the subdimensions of disrupted parenting suggested that parents who experienced more PTS symptoms showed more *role/boundary confusion*, and more *fearful/disoriented* parenting behavior. The relation with these specific dimensions of disrupted parenting could lie in the nature of the PTS symptoms. Typically, PTS symptoms cause a high burden on parents’ functioning (Jellestad et al., 2021), which might cause them (unconsciously) to seek support from their children in dealing with their symptoms, resulting in behaviors such as *demanding affection* or *seeking attention* from their children, which are typical role confusion behaviors. In the same way, symptoms such as hypervigilance or startling behavior, which are typical PTS symptoms (American Psychiatric Association, 2022), might directly be visible in parenting behavior. For example, parents might be *startled by their children without a clear cause*, or they might exhibit *vigilant postures in interaction with their children*, which are typical fearful/disoriented behaviors. These patterns warrant attention, as they could severely impact children's internal working model of their caregiver, if persisting (Lyons‐Ruth et al., 1999). Ultimately, this could result in a disorganized attachment relationship with the caregiver, where the parent is not a source of comfort but rather a source of distress for the child (Lyons‐Ruth & Spielman, 2004).

Further exploratory analyses showed no meaningful relations between childhood maltreatment experiences and subdimensions of disrupted parenting nor for mediation of PTS symptoms in this relation. There was some initial evidence for a negative relation between childhood maltreatment and *intrusiveness/negativity*, and possible mediation of PTS symptoms in the relation between childhood maltreatment and *role/boundary confusion* and *fearful/disoriented behaviors*. The nature of these relations is unclear yet, however, as these results only showed under the diffuse distribution, or the size was negligible in the current sample. Therefore, those explorative findings do not allow firm conclusions but could still provide valuable input for future research.

The current study used Bayesian regression with informed prior probability distributions that quantitively reflect our beliefs about the parameters of interest and the uncertainty surrounding these beliefs. Despite the fact that these priors were formulated based on a systematic literature search and thus reflect the present knowledge about the parameters, the observed data did not match these priors well in all estimated relations. The data fit the prior distribution well in estimating the relations between childhood maltreatment experiences and PTS symptoms and between PTS symptoms and disrupted parenting behavior, but it did not for the relation between childhood maltreatment experiences and disrupted parenting. This was indicated by the fact that the data fit best under the diffuse prior distribution, so adding an informed prior did not improve the fit of the model (Kruschke et al., 2012). An explanation might be that the prior was informed with earlier findings including disrupted parenting as outcome, and studies were included if they incorporated at least one form of disrupted parenting (such as withdrawal or intrusiveness). As a result, various forms of negative parenting behavior, measured with various measurement systems, were included. We may have wrongly assumed that these subtypes of disrupted parenting, when taken together, would form a well‐informed prior estimation for the overall construct of disrupted parenting. In reality, not all of the included forms of negative parenting may correlate strongly enough with the overall construct of disrupted parenting. With that, this study does not only show the benefits of Bayesian estimation, when priors improve model fit, but also shows the importance of checking prior sensitivity, to ensure suitable conclusions even when data do not align with the prior distributions (Zondervan‐Zwijnenburg et al., 2017).

### 4.1 Limitations

The current study could be limited by the small sample size, of just 52 participants. Generally, mediation analysis in such small samples is discouraged, and therefore we used Bayesian statistics, which is the advised alternative (Koopman et al., 2015). Still, the small sample can have affected the results, as it could explain why the results using the different priors did not always lead to the same conclusions. In smaller samples, results can be affected by the prior specification, as the influence of the data itself is relatively small (compared to a larger sample) (Van de Schoot et al., 2015). A larger sample size could have improved the representativity and generalizability of the results, so that the results would possibly align more with previous findings. At the same time, the sample that was recruited is from a very specific, highly clinical population that experiences lots of complex stressors and lives in challenging circumstances, what could make them ‘hard to reach’ for research (Ellard‐Gray et al., 2015). Therefore, we also see it as a strength that the current study can contribute to the existing evidence that is available about this target population.

This study may further be limited by the setting that was used, especially with regard to the video paradigm. Where many studies use the strange situation procedure or other paradigms that use separation/reunion elements that activate the attachment system of children (and thus require the parent to actively support the child) to code disrupted parenting behavior (see e.g., Ensink et al., 2016), we used a shorter, less intensive paradigm where parents and children played with each other for 7 min, as was also used in other studies (e.g., Guyon‐Harris et al., 2022; Madigan, Moran, et al., 2006). The free‐play paradigm may have been too mild to evoke withdrawing parenting behavior, as this occurred so little that reliability for this dimension was not high enough. At the same time, there is no evidence that a separation/reunion episode, such as used in the strange situation procedure, would be better to measure disrupted parenting behavior (Madigan, Bakermans‐Kranenburg, et al., 2006). It could thus also be possible that withdrawing parenting behavior was hardly present in the current sample, as others, using a similar paradigm, *could* reliably code withdrawing parenting behavior (Guyon‐Harris et al., 2021), and did find support for an association in the proposed direction (Burtchen et al., 2022). Furthermore, a separation/reunion setting was hard to establish in the current study, for example because research appointments occurred in the home, and then the parent leaving the room might be so normal for the child that it is not stressful. As disrupted parenting in general, and most of the different subdimensions, could still reliably be coded, we believe that the current study still gives a representative insight in disrupted parenting in this sample.

### 4.2 Implications

Based on the current study, attention is warranted to the complex interplay between childhood maltreatment experiences, PTS symptoms, and disrupted parenting behavior. The relation between childhood maltreatment and disrupted parenting via PTS symptoms was negligible in the current study, but it should be noted that the sample under study experienced a multitude of childhood maltreatment experiences and other traumatic experiences, PTS symptoms and disrupted parenting behaviors, that are likely to cause a high burden on an already vulnerable group. The current study shows that childhood maltreatment experiences may still be associated with the experience of PTS symptoms later in life, even in the aftermath of severe IPV. This implies that practitioners should not only focus on PTS symptoms that are related to the recent IPV experiences but should also take the impact of childhood maltreatment experiences into account. No causal conclusions can be drawn from the results, however, as data were cross‐sectional in nature, and alternative explanations cannot be ruled out.

In addition, the finding that PTS symptoms seemed to be related to parenting behavior, more specifically *role/boundary confusion* and *fearful/disoriented behaviors*, points to the relevance of systemic assessment and intervention, where parents who struggle with PTS symptoms are also observed in their parenting practices and receive necessary support to maintain healthy relationships with their children. A relevant question for future studies could then be whether this parenting behavior improves after treatment of parents’ PTS symptoms. Also, apart from these findings, this paper shows the need for effective intervention in this population. Almost all mothers experienced childhood maltreatment, the average level of PTS symptoms was in the clinical range and half of the sample was classified as showing disrupted parenting behavior. Targeted support on these three levels could substantially improve the lives of mothers and children and could help to end the cycle of trauma and violence across generations.

## FUNDING INFORMATION

This study was funded by ZonMw (The Netherlands Organization for Health Research and Development), project number 1026001 1910001. ZonMw monitored the project through yearly reports and evaluations. Additional financial support was provided by the Institute of Education and Child Studies, Leiden University. Both funding sources had no substantive involvement (i.e., in study design, in collection, analysis and interpretation of data in the study, in the writing of the report and in the decision to submit the article for publication).

## CONFLICT OF INTEREST STATEMENT

The authors declare no conflicts of interest.

## Supporting information



Supporting Information 1: Appendix A: Overview of the Systematic Literature Search

Supporting Information 2: Appendix B: Results of the Additional Explorative Analyses

## Data Availability

Raw data include sensitive participant information, and can therefore not be shared. R‐scripts of the analyses, and the data extraction sheet for the systematic literature search will be made publicly available at the Open Science Framework repository (DOI: 10.17605/OSF.IO/9EW7P), after publication of the manuscript.
