# Supplementary material for: Childhood maltreatment and disrupted parenting in mothers exposed to intimate partner violence: Mediation via mothers' posttraumatic stress symptoms?
Source: Infant Ment Health J. 2026 Aug 13;47(5):e70114. doi: 10.1002/imhj.70114 (PMC13470465; doi:10.1002/imhj.70114)
Supplement: Supplementary file 1 [file IMHJ-47-0-s001.docx]

**Appendix A: Overview of the Systematic Literature Search**

Prior elicitation was based on a systematic review, with the goal to specify the prior distribution of the slope for the different pathways in the mediation model under study. A literature search was carried out in the database ‘Web of Science’. The search was intended to grasp the relevant subset of the available literature on the relation between parental childhood maltreatment experiences, parental post traumatic stress (PTS) symptoms, and disruptive parenting behavior. The articles were screened on topic and on sample characteristics. Search terms were specified for sample and topic, and are depicted in Table A1.

**Table A1**

*Sample Characteristics and Topic Terms that were used for the Literature Search*

| Sample characteristics | Parent* OR mother OR father OR maternal OR paternal  AND |
| --- | --- |
| Topic terms | Child OR childhood AND (maltreat* OR adverse experiences OR trauma OR abuse OR neglect OR domestic violence OR IPV OR intimate partner violence OR interparental violence)  AND  posttraumatic stress disorder OR PTSD OR trauma OR traumatic stress  AND  disruptive OR frightening OR frightened OR harsh OR intrusive OR controlling OR coercive OR hostil* OR withdraw* OR reject* OR negative OR negative OR overreactive* OR harsh discipline OR overprotect* OR authorit*  AND  Caregiving OR parenting |

**Selection of Studies**

A total of 904 studies resulted from the initial search. All studies were uploaded in ASReview for screening. This is a machine learning tool for screening and selecting articles (Van de Schoot et al., 2021). The tool works in such a way that studies are ranked by relevance, so that the most relevant articles are showed first. The relevance is estimated based on an algorithm, that is composed on the basis of articles that are deemed relevant and irrelevant, and is adapted after every screened article. The advantage of ASReview is that it can increase efficiency, because not all articles have to be screened manually (Van de Schoot et al., 2021). For this review, a stopping rule of 100 articles was chosen, meaning that screening was stopped after 100 studies consecutively were deemed irrelevant.

One researcher screened the articles for an effect size of one or more of the paths of the mediation model that are the focus of the current study (childhood maltreatment 🡪 disruptive parenting, childhood maltreatment 🡪 PTS symptoms, and PTS symptoms 🡪 disruptive parenting). Screening was performed on the following inclusion criteria: studies (a) had to be published in peer-reviewed journals and available in English, (b) the focus of the studies was on parents, (c) the age of the children, if they were included, was between 0-6 years old, (d) parenting behavior, if reported on, was measured through observation. Furthermore, the review focused specifically on forms of negative parenting behavior. The abstracts were screened first. If the abstract did not include enough information to decide whether the inclusion criteria were met, the full text article was screened. A total of 384 articles were screened, from which 35 eligible papers were extracted. The remaining studies were automatically excluded by ASReview. To be sure that enough relevant papers were included in the final set, recent reviews and a meta-analysis (Christie et al., 2019; Hughes & Cossar, 2015; Lotto et al., 2023; Savage et al., 2019) were checked for additional studies. This resulted in four additional relevant articles, which brought the total on 39 articles.

More extensive screening of the eligible articles revealed that 5 articles did not report sufficient statistics to be included, and could thus not be used for estimating the priors. These were papers that did not include an effect size estimate and/or p value (*n* = 2), or papers that reported effect sizes that were part of a multivariate model (unless there was controlled for ≤ 2 demographic characteristics) (*n* = 2). Furthermore, 12 articles reported about the same four samples in a likewise model. In these cases, the study that reported on the largest sample was included, unless this paper did not include the best estimate for one or more of the paths under study. This was based on a) the type of measurement level that was analyzed (if one study reported about a categorical measure and the other only about interval measures, the one with interval measures was chosen), b) the number of pathways that were analyzed (the study that reported on the most pathways was chosen) and c), the number of measures (if one article included results on one form of negative parenting behavior and another included results on more relevant forms, the later was used). Four of these 12 articles were thus used for prior elicitation, the other eight were excluded.

This resulted in a final set of 27 articles that could be used for prior elicitation. Within these articles, effect sizes other than correlation coefficients (e.g. an F-statistic or unstandardized regression coefficient) were converted to a correlation coefficient (*n* = 3). Additionally, effect sizes were all defined in the same direction (i.e. an effect size that related to a concept such as ‘non-hostility’ was converted so that it related to the concept ‘hostility’). Furthermore, if studies reported more than one estimate for a pathway (e.g. if multiple forms of negative parenting behavior or child maltreatment were analyzed separately), the different estimates were aggregated. Altogether the articles then provided 30 correlation coefficients.

**Specification of High Risk Samples**

As the sample of the current study (i.e., the sample over which the posterior distribution is drawn) is characterized by demographic risks (in addition to the IPV-exposure), with most parents being single and unemployed, and few parents having completed higher education (see Table 1), we decided to specify a separate prior based on the samples with a high level of demographic risks. High risk samples for the current study were characterized as samples in which at least one of the factors was apparent, for at least 50% of the sample: (1) unemployed, (2) low income, (3) low level of education, (4) living apart from the child’s other parent OR at least 50% of the included parents were exposed to intimate partner violence in their adult life.

The final set of articles that was used for prior elicitation is shown in Table A2. A data extraction sheet with additional information on the articles that were included and excluded in the study can be found at the Open Science Framework (BLINDED FOR REVIEW).

A total of three priors were specified for each pathway, one prior was specified based on the high risk samples, and was thought to be most informative for the current posterior distribution. Another, less informative prior was specified for all samples taken together (both the high risk and the non high-risk). Lastly, a diffuse or non-informative prior was specified. The reason the last two priors were specified was to allow for a sensitivity analysis of the most informed prior.

**Table A2**

*Articles included in the Systematic Literature Search*

| Authors (year) | Pathway of the mediation model | Risk level |
| --- | --- | --- |
| Ammerman et al. (2012) | Childhood maltreatment – PTS symptoms | High |
| Bailey et al. (2012) | Childhood maltreatment – parenting | High |
| Brockman et al. (2016) | PTS symptoms – parenting | Not high |
| Burtchen et al. (2022) | Childhood maltreatment – parenting | High |
| Cheng et al. (2024) | PTS symptoms – parenting | Not high |
| Ensink et al. (2016) | Childhood maltreatment – parenting | Not high |
| Franz et al. (2022) | PTS symptoms – parenting | Not high |
| Greene et al. (2021) | Childhood maltreatment – PTS symptoms | High |
| Guyon Harris et al. (2020) | Childhood maltreatment – parenting | High |
| Hicks and Dayton (2019) | Childhood maltreatment – PTS symptoms | High |
| Hinesley et al. (2020) | Childhood maltreatment – PTS symptoms | Not high |
| Julian et al. (2019) | Childhood maltreatment – parenting | Not high |
| Liu et al. (2021) | Childhood maltreatment – parenting | Not high |
| Lyons-Ruth and Block (1996) | Childhood maltreatment – symptoms  Childhood maltreatment – parenting  PTS symptoms – parenting | High |
| MacMillan et al. (2021) | Child maltreatment – parenting | Not high |
| Narayan et al. (2017) | Childhood maltreatment – parenting | High |
| Pasalich et al. (2016) | Childhood maltreatment – parenting | High |
| Penner et al. (2023) | Childhood maltreatment – PTS symptoms | Not high |
| Schechter et al. (2004) | Childhood maltreatment – PTS symptoms | High |
| Schechter et al. (2010) | PTS symptoms – parenting | High |
| Sexton et al. (2017) | Childhood maltreatment – parenting | Not high |
| Shin et al. (2024) | Childhood maltreatment – parenting | High |
| Stacks et al. (2014) | Childhood maltreatment – PTS symptoms  Childhood maltreatment – parenting  PTS symptoms – parenting | Not high |
| Torrisi et al. (2018) | PTS symptoms – parenting | High |
| Van Ee et al. (2013) | PTS symptoms – parenting | High |
| Webster-Stratton and Hammond (1988) | Childhood maltreatment – parenting | Not high |
| Zvara et al. (2015) | Childhood maltreatment – parenting | High |

**References**

Ammerman, R. T., Putnam, F. W., Chard, K. M., Stevens, J., & Van Ginkel, J. B. (2012). PTSD in depressed

mothers in home visitation. *Psychological Trauma: Theory, Research, Practice, and Policy*, *4*(2), 186. <https://doi.org/10.1037/a0023062>

Bailey, H. N., DeOliveira, C. A., Wolfe, V. V., Evans, E. M., & Hartwick, C. (2012). The impact of childhood

maltreatment history on parenting: A comparison of maltreatment types and assessment methods. *Child Abuse & Neglect*, *36*(3), 236-246. <https://doi.org/10.1016/j.chiabu.2011.11.005>

Brockman, C., Snyder, J., Gewirtz, A., Gird, S. R., Quattlebaum, J., Schmidt, N., Pauldine, M.R.,

Schrepferman, L., Hayes, C., & DeGarmo, D. (2016). Relationship of service members’

deployment trauma, PTSD symptoms, and experiential avoidance to postdeployment family

reengagement. *Journal of Family Psychology*, *30*(1), 52. <https://doi.org/10.1037/fam0000152>

Burtchen, N., Alvarez-Segura, M., Urben, S., Giovanelli, C., Mendelsohn, A. L., Guedeney, A., & Schechter,

D. S. (2022). Effects of maternal trauma and associated psychopathology on atypical maternal behavior and infant social withdrawal six months postpartum. *Attachment & Human Development*, *24*(6), 750-776. <https://doi.org/10.1080/14616734.2022.2142894>

Cheng, C. H., Ali‐Saleh Darawshy, N., Lee, S., Brigman, H., DeGarmo, D., & Gewirtz, A. (2024). Replication

and extension of the military family stress model: The after deployment adaptive parenting tools ADAPT4U study. *Family Process*, *63*(1), 80-96. <https://doi.org/10.1111/famp.12918>

Ensink, K., Bégin, M., Normandin, L., & Fonagy, P. (2016). Maternal and child reflective functioning in the

context of child sexual abuse: Pathways to depression and externalising difficulties. *European Journal of Psychotraumatology*, *7*(1), 30611. <https://doi.org/10.3402/ejpt.v7.30611>

Franz, M. R., Kumar, S. A., Brock, R. L., Calvi, J. L., & DiLillo, D. (2022). Parenting behaviors of mothers

with posttraumatic stress: The roles of cortisol reactivity and negative emotion. *Journal of Family Psychology*, *36*(1), 130. <https://doi.org/10.1037/fam0000865>

Greene, C. A., Wesley, A. L., McCarthy, K. J., Anderson, E. L., Wakschlag, L. S., & Briggs-Gowan, M. J.

(2021). Maternal socialization is associated with prosocial behavior in at-risk young children. *Vulnerable Children and Youth Studies*, *16*(1), 7-19. <https://doi.org/10.1080/17450128.2020.1861399>

Guyon-Harris, K. L., Madigan, S., Bronfman, E., Romero, G., & Huth-Bocks, A. C. (2021). Prenatal

identification of risk for later disrupted parenting behavior using latent profiles of childhood maltreatment. *Journal of Interpersonal Violence*, *36*(23-24), NP13517-NP13540. <https://doi.org/10.1177/0886260520906175>

Hicks, L. M., & Dayton, C. J. (2019). Mindfulness and trauma symptoms predict child abuse potential in

risk-exposed, men and women during pregnancy. *Child Abuse & Neglect*, *90*, 43-51. <https://doi.org/10.1016/j.chiabu.2019.01.018>

Hinesley, J., Amstadter, A., Sood, A., Perera, R. A., Ramus, R., & Kornstein, S. (2020). Adverse childhood

experiences, maternal/fetal attachment, and maternal mental health. *Women's Health Reports*, *1*(1), 550-555. <https://doi.org/10.1089/whr.2020.0085>

Julian, M. M., King, A. P., Bocknek, E. L., Mantha, B., Beeghly, M., Rosenblum, K. L., & Muzik, M. (2019).

Associations between oxytocin receptor gene (OXTR) polymorphisms, childhood trauma, and parenting behavior. *Developmental Psychology*, *55*(10), 2135. <https://doi.org/10.1037/dev0000783>

Liu, S., Dong, S., & Wang, Z. (2021). Maternal history of childhood maltreatment and control strategies: A

search for mechanisms. *Journal of Marriage and Family*, *83*(4), 1099-1115. <https://doi.org/10.1111/jomf.12751>

Lyons‐Ruth, K., & Block, D. (1996). The disturbed caregiving system: Relations among childhood trauma,

maternal caregiving, and infant affect and attachment. *Infant Mental Health Journal: Official Publication of The World Association for Infant Mental Health*, *17*(3), 257-275. [https://doi.org/10.1002/(SICI)1097-0355(199623)17:3<257::AID-IMHJ5>3.0.CO;2-L](https://doi.org/10.1002/(SICI)1097-0355(199623)17:3%3c257::AID-IMHJ5%3e3.0.CO;2-L)

MacMillan, K. K., Lewis, A. J., Watson, S. J., Jansen, B., & Galbally, M. (2021). Maternal trauma and

emotional availability in early mother-infant interaction: Findings from the Mercy Pregnancy and Emotional Well-being Study (MPEWS) cohort. *Attachment & Human Development*, *23*(6), 853-875. <https://doi.org/10.1080/14616734.2020.1790116>

Narayan, A. J., Kalstabakken, A. W., Labella, M. H., Nerenberg, L. S., Monn, A. R., & Masten, A. S. (2017).

Intergenerational continuity of adverse childhood experiences in homeless families: Unpacking exposure to maltreatment versus family dysfunction. *American Journal of Orthopsychiatry*, *87*(1), 3. <https://doi.org/10.1037/ort0000133>

Pasalich, D. S., Cyr, M., Zheng, Y., McMahon, R. J., & Spieker, S. J. (2016). Child abuse history in teen

mothers and parent–child risk processes for offspring externalizing problems. *Child Abuse & Neglect*, *56*, 89-98. <https://doi.org/10.1016/j.chiabu.2016.04.011>

Penner, F., Khoury, J. E., Enlow, M. B., & Lyons-Ruth, K. (2023). Threat versus deprivation in mother's

childhood: differential relations to hair cortisol and psychopathology in pregnancy. *Child Abuse & Neglect*, *139*, 106107. <https://doi.org/10.1016/j.chiabu.2023.106107>

Schechter, D. S., Zeanah Jr, C. H., Myers, M. M., Brunelli, S. A., Liebowitz, M. R., Marshall, R. D., Coates,

S.W., Trabka, A.K., Baca, P., & Hofer, M. A. (2004). Psychobiological dysregulation in violence-exposed mothers: Salivary cortisol of mothers with very young children pre-and post-separation stress. *Bulletin of the Menninger Clinic*, *68*(4), 319-336. <https://doi.org/10.1521/bumc.68.4.319.56642>

Schechter, D. S., Willheim, E., Hinojosa, C., Scholfield-Kleinman, K., Turner, J. B., McCaw, J., Zeanah, C.H.,

& Myers, M. M. (2010). Subjective and objective measures of parent-child relationship

dysfunction, child separation distress, and joint attention. *Psychiatry*, *73*(2), 130-144.

<https://doi.org/10.1521/psyc.2010.73.2.130>

Sexton, M. B., Davis, M. T., Menke, R., Raggio, G. A., & Muzik, M. (2017). Mother–child interactions at six

months postpartum are not predicted by maternal histories of abuse and neglect or maltreatment type. *Psychological Trauma: Theory, Research, Practice, and Policy*, *9*(5), 622. <https://doi.org/10.1037/tra0000272>

Shin, J. Y., McMahon, T. J., Penner, F., & Lowell, A. (2024). Maternal childhood trauma, caregiving

behavior, and child cognitive development in the context of drug addiction. *American Journal of Orthopsychiatry*. <https://doi.org/10.1037/ort0000802>

Stacks, A. M., Muzik, M., Wong, K., Beeghly, M., Huth-Bocks, A., Irwin, J. L., & Rosenblum, K. L. (2014).

Maternal reflective functioning among mothers with childhood maltreatment histories: Links to sensitive parenting and infant attachment security. *Attachment & Human Development*, *16*(5), 515-533. <https://doi.org/10.1080/14616734.2014.935452>

Torrisi, R., Arnautovic, E., Perizzolo, V. P., Vital, M., Manini, A., Suardi, F., Gex-Fabry, M., Rusconi Serpa, S.,

& Schechter, D. S. (2018). Developmental delay in communication among toddlers and its

relationship to caregiving behavior among violence-exposed, posttraumatically stressed

mothers. *Research in Developmental Disabilities*, *82*, 67-78.

<https://doi.org/10.1016/j.ridd.2018.04.008>

Van Ee, E., Sleijpen, M., Kleber, R. J., & Jongmans, M. J. (2013). Father‐involvement in a refugee sample:

Relations between posttraumatic stress and caregiving. *Family Process*, *52*(4), 723-735.

<https://doi.org/10.1111/famp.12045>

Webster-Stratton, C., & Hammond, M. (1988). Maternal depression and its relationship to life stress,

perceptions of child behavior problems, parenting behaviors, and child conduct problems. *Journal of Abnormal Child Psychology*, *16*(3), 299-315. <https://doi.org/10.1007/BF00913802>

Zvara, B. J., Mills-Koonce, W. R., Carmody, K. A., Cox, M., & Family Life Project Key Investigators. (2015).

Childhood sexual trauma and subsequent parenting beliefs and behaviors. *Child Abuse & Neglect*, *44*, 87-97. <https://doi.org/10.1016/j.chiabu.2015.01.012>
