# Supplementary material for: Childhood maltreatment and disrupted parenting in mothers exposed to intimate partner violence: Mediation via mothers' posttraumatic stress symptoms?
Source: Infant Ment Health J. 2026 Aug 13;47(5):e70114. doi: 10.1002/imhj.70114 (PMC13470465; doi:10.1002/imhj.70114)
Supplement: Supplementary file 2 [file IMHJ-47-0-s002.docx]

**Appendix B – Results of the Additional Explorative Analyses**

**Table B1**

*Relation between Childhood Maltreatment and Dimensions of Disrupted Parenting*

| Type of prior | Predictor | | | *β* | | *SD* | *95% PPI* | | | | *% in ROPE* | |
| --- | --- | --- | --- | --- | --- | --- | --- | --- | --- | --- | --- | --- |
|  |  |  |  |  |  |  | *LL* | | *UL* | |  |  |
| Childhood maltreatment 🡪 Affective communication errors | | | | | | | | | | |  | |
| Informative | Childhood maltreatment  Age child | .05  -.19 | | | | .08  .08 | -.10  -.34 | | .22  -.04 | | 77.03  9.51 | |
| Weakly informative | Childhood maltreatment | .01 | | | | .09 | -.17 | | .18 | | 76.85 | |
|  | Age child | -.19 | | | | .08 | -.34 | | -.04 | | 9.60 | |
| Diffuse^a^ | Childhood maltreatment | -.13 | | | | .13 | -.39 | | .13 | | 38.02 | |
|  | Age child | -.20 | | | | .07 | -.35 | | -.05 | | 7.26 | |
| Childhood maltreatment 🡪 Role/boundary confusion | | | | | | | | | | |  | |
| Informative | Childhood maltreatment | .06 | | | | .08 | -.10 | | .22 | | 69.66 | |
|  | Age child | -.15 | | | | .08 | -.31 | | .00 | | 23.13 | |
| Weakly informative | Childhood maltreatment | .01 | | | | .09 | -.17 | | .19 | | 76.22 | |
|  | Age child | -.16 | | | | .08 | -.31 | | -.01 | | 21.75 | |
| Diffuse | Childhood maltreatment | -.13 | | | | .13 | -.39 | | .14 | | 39.67 | |
|  | Age child | -.16 | | | | .08 | -.31 | | -.01 | | 18.41 | |
| Childhood maltreatment 🡪 Fearful/disoriented behavior | | | | | | | | | | |  | |
| Informative | Childhood maltreatment | .03 | | | | .08 | -.13 | | .19 | | 78.56 | |
|  | Age child | -.19 | | | | .08 | -.34 | | -.04 | | 9.99 | |
| Weakly informative | Childhood maltreatment | -.03 | | | | .09 | -.20 | | .15 | | 74.29 | |
|  | Age child | -.19 | | | | .08 | -.34 | | -.04 | | 9.14 | |
| Diffuse^a^ | Childhood maltreatment | -.20 | | | | .13 | -.46 | | .06 | | 19.76 | |
|  | Age child | -.20 | | | | .07 | -.34 | | -.05 | | 6.43 | |
| Childhood maltreatment 🡪 Intrusiveness/negativity | | | | | | | | | | |  | |
| Informative | Childhood maltreatment | | -.02 | | .08 | | | -.18 | | .15 | | 80.46 |
|  | Age child | | -.21 | | .07 | | | -.35 | | -.06 | | 5.06 |
| Weakly informative | Childhood maltreatment | | -.08 | | .09 | | | -.26 | | .10 | | 56.74 |
|  | Age child | | -.21 | | .07 | | | -.35 | | -.07 | | 4.00 |
| Diffuse | Childhood maltreatment | | -.31 | | .13 | | | -.55 | | -.06 | | 2.81 |
|  | Age child | | -.22 | | .07 | | | -.36 | | -.08 | | 1.55 |

^a^reported for the model with 40000 iterations.

**Table B2**

*Relation between PTS symptoms and Dimensions of Disrupted Parenting*

| Type of prior | Predictor | | | *β* | | *SD* | *95% PPI* | | | | *% in ROPE* | |
| --- | --- | --- | --- | --- | --- | --- | --- | --- | --- | --- | --- | --- |
|  |  |  |  |  |  |  | *LL* | | *UL* | |  |  |
| PTS symptoms 🡪 Affective communication errors | | | | | | | | | | |  | |
| Informative | PTS symptoms  Age child | .18  -.20 | | | | .09  .08 | .00  -.35 | | .37  -.05 | | 18.32  6.63 | |
| Weakly informative | PTS symptoms | .15 | | | | .10 | -.04 | | .33 | | 29.68 | |
|  | Age child | -.20 | | | | .08 | -.35 | | -.05 | | 6.77 | |
| Diffuse | PTS symptoms | .04 | | | | .13 | -.22 | | .31 | | 55.16 | |
|  | Age child | -.19 | | | | .08 | -.34 | | -.04 | | 8.80 | |
| PTS symptoms 🡪 Role/boundary confusion | | | | | | | | | | |  | |
| Informative | PTS symptoms | .23 | | | | .10 | .04 | | .42 | | 6.71 | |
|  | Age child | -.17 | | | | .08 | -.32 | | -.02 | | 14.95 | |
| Weakly informative^a^ | PTS symptoms | .20 | | | | .09 | .01 | | .38 | | 13.16 | |
|  | Age child | -.17 | | | | .08 | -.32 | | -.02 | | 15.79 | |
| Diffuse | PTS symptoms | .14 | | | | .13 | -.13 | | .41 | | 36.73 | |
|  | Age child | -.17 | | | | .08 | -.32 | | -.02 | | 17.53 | |
| PTS symptoms 🡪 Fearful/disoriented behavior | | | | | | | | | | |  | |
| Informative | PTS symptoms | .22 | | | | .09 | .04 | | .41 | | 7.96 | |
|  | Age child | -.21 | | | | .08 | -.36 | | -.06 | | 5.57 | |
| Weakly informative^a^ | PTS symptoms | .19 | | | | .09 | .00 | | .37 | | 15.25 | |
|  | Age child | -.20 | | | | .08 | -.35 | | -.06 | | 6.04 | |
| Diffuse | PTS symptoms | .13 | | | | .13 | -.14 | | .38 | | 39.41 | |
|  | Age child | -.20 | | | | .08 | -.35 | | -.05 | | 7.28 | |
| PTS symptoms 🡪 Intrusiveness/negativity | | | | | | | | | | |  | |
| Informative | PTS symptoms | | .14 | | .10 | | | -.05 | | .33 | | 33.83 |
|  | Age child | | -.22 | | .08 | | | -.37 | | -.07 | | 3.98 |
| Weakly informative^a^ | PTS symptoms | | .11 | | .09 | | | -.08 | | .30 | | 46.78 |
|  | Age child | | -.21 | | .08 | | | -.36 | | -.06 | | 4.11 |
| Diffuse^a^ | PTS symptoms | | -.04 | | .13 | | | -.30 | | .22 | | 56.54 |
|  | Age child | | -.20 | | .08 | | | -.35 | | -.06 | | 6.26 |

^a^reported for the model with 40000 iterations.

**Table B3**

*Indirect effects of Childhood Maltreatment via PTS symptoms on subdimensions of Disrupted Parenting*

| Type of prior | *β* | *SD* | *95% PPI* | | *% in ROPE* | |
| --- | --- | --- | --- | --- | --- | --- |
|  |  |  | *LL* | *UL* |  |  |
| Outcome: Affective communication errors |  |  |  |  | |  |
| Informative | .05 | .03 | .00 | -.12 | | 94.78 |
| Weakly informative | .04 | .03 | -.01 | .12 | | 97.28 |
| Diffuse | .02 | .04 | -.05 | .11 | | 98.61 |
| Outcome: Role/boundary confusion |  |  |  |  | |  |
| Informative | .07 | .03 | .01 | .14 | | 87.29 |
| Weakly informative | .06 | .04 | .00 | .14 | | 87.89 |
| Diffuse | .04 | .05 | -.02 | .15 | | 90.47 |
| Fearful/disoriented behaviors |  |  |  |  | |  |
| Informative^a^ | .06 | .03 | .01 | .14 | | 88.28 |
| Weakly informative | .06 | .04 | .00 | .14 | | 88.52 |
| Diffuse | .05 | .04 | -.02 | .15 | | 90.26 |
| Instrusiveness/negativity |  |  |  |  | |  |
| Informative | .04 | .03 | -.01 | .11 | | 98.54 |
| Weakly informative | .04 | .03 | -.02 | .11 | | 98.74 |
| Diffuse | .01 | .04 | -.06 | .09 | | 100 |

^a^reported for the model with 40000 iterations.
